# Prevalence of Cryptosporidium and Giardia infections in under-five children with diarrhoea in Blantyre, Malawi

**DOI:** 10.1186/s12879-024-08979-w

**Published:** 2024-01-09

**Authors:** Joseph E. V. Bitilinyu-Bangoh, Samra Riesebosch, Marije Rebel, Paul Chiwaya, Sjoerd P. Verschoor, Wieger P. Voskuijl, Henk D. F. H. Schallig

**Affiliations:** 1 Department of Medical Microbiology and Infection Prevention, Laboratory for Experimental Parasitology, Amsterdam University Medical Centre, Amsterdam Institute for Infection and Immunity, Meibergdreef 9, 1105 AZ Amsterdam, The Netherlands; 2 Department of Paediatrics and Child Health, Kamuzu University for Health Sciences, Blantyre, Malawi; 3 Amsterdam University Medical Centre, Amsterdam Institute for Global Child Health, Emma Children’s Hospital, Meibergdreef 9, 1105 AZ Amsterdam, The Netherlands; 4 Amsterdam Institute for Global Health and Development, Amsterdam University Medical Centre, Meibergdreef 9, 1105 AZ Amsterdam, The Netherlands; 5 Department of Paediatrics and Child Health, Kamuzu University of Health Sciences, Blantyre, Malawi; 6 Public Health Institute of Malawi, Lilongwe, Malawi; 7 Department Experimental Immunology, Amsterdam University Medical Centre, Amsterdam, The Netherlands; 8 University of Malawi, Zomba, Malawi; 9 Training Institute Global Health and Tropical Medicine (OIGT), Utrecht, The Netherlands

**Keywords:** *Cryptosporidium*, *Giardia*, Diarrhoea, Diagnosis, Prevalence, Malawi

## Abstract

**Background:**

Diarrhoeal diseases are common among children in low- and middle-income countries and are major causes of morbidity and mortality. *Cryptosporidium* and *Giardia* are considered to be the main parasitic causes of diarrhoea in children. The aim of the present study was to determine the prevalence and associated factors of *Cryptosporidium* and *Giardia* infection in children under five years of age presenting at two health centres (Ndirande and Limbe) in Blantyre, Malawi.

**Methods:**

This cross-sectional study was performed from February to July 2019 and included 972 children under 5 years of age with diarrhoea. Stool samples were immediately tested after collection at enrolment with a rapid diagnostic test for *Cryptosporidium* and *Giardia* infection. Descriptive statistics were used to assess the prevalence of these protozoan parasitic infections, and differences in the basic demographic and anthroponotic variables (between children with diarrhoea and parasite infection, being either *Cryptosporidium* and *Giardia* or both versus children with diarrhoea but no RDT confirmed parasite infection) were assessed. Their association with *Cryptosporidium* and *Giardia* infection was analysed using simple logistic regressions.

**Results:**

Of the children recruited, 88 (9.1%) tested positive for *Cryptosporidium* and 184 (18.9%) for *Giardia*. Children with only a *Giardia* infection or a coinfection (of both parasites) were significantly older (mean age 24–26 months) compared to children with only a *Cryptosporidium* infection (mean age 13 months) or no parasitic infection (mean age 14 months). No significant differences were found with respect to gender, body temperature, stunting or wasting between the different groups of children with moderate to severe diarrhoea. Children attending the Ndirande health centre had almost two times higher odds of testing positive for both infections than those attending Limbe health centre.

**Conclusion:**

*Cryptosporidium* and *Giardia* infections are highly prevalent in children < 5 years with moderate to severe diarrhoea attending the Limbe and Ndirande health centres in Blantyre, Malawi.

## Background

Diarrhoea is one of the most important causes of morbidity and mortality in children younger than the age of 5 years (< 5 years) in low- and middle-income countries (LMICs) [1]. There are approximately 1.7 billion cases of childhood diarrhoea disease every year, resulting in the death of approximately 525 000 children under five [2, 3], making diarrhoea the third leading cause of death in < 5 years. Diarrhoea is also a leading cause of malnutrition in young children, and malnutrition in turn is a major factor for the development of diarrhoea, a malicious cycle often difficult to interrupt [4].

Gastro-intestinal infections with protozoan parasites, such as *Cryptosporidium* and *Giardia* species are, next to Rotavirus, important causative agents of childhood diarrhoea [5, 6]. Vaccination against Rotavirus has shown to be very effective in reducing disease caused by this pathogen [7, 8], but this strategy is presently not available for protozoan parasites. Therefore, diagnosis and supportive treatment are cornerstone for the management of diarrhoea disease in young children. Infections with *Giardia* are treated with metronidazole in our setting, although treatment-refractory giardiasis is being reported [9]. In contrast, for *Cryptosporidium* no effective treatment is currently available, albeit that the use of nitazoxanide is suggested in some particular circumstances [10]. Diarrhoea is further managed by providing supportive treatment comprising oral rehydration solution (ORS), in combination with a 10–14 day supplemental treatment course of dispersible 20 mg zinc tablets, which shortens diarrhoea duration and improves outcomes [11].

Quick, accurate and cost effective diagnosis of diarrhoea (caused by intestinal parasites) is important to properly manage infected children and to timely start appropriate treatment [5, 6]. The diagnosis of infections with intestinal parasites is traditionally performed by microscopic examination of stool samples, which might be cumbersome, time-consuming and prone to errors [12]. Rapid diagnostic tests (RDTs) provide an attractive alternative by combining sufficient diagnostic accuracy with speed and ease of operation [13].

Furthermore, the implementation of rotavirus vaccination and other public health interventions has led to a worldwide declining trend over time in < 5 years mortality and diarrhoea-related deaths [14]. However, in Malawi there is a continued environmental vulnerability to diarrhoea in < 5 years, as analysis of the 2017 Demographic and Health Survey in Malawi revealed an increase of children under the age of five with diarrhoea since 2010 [15]. Adequate knowledge about the epidemiology and factors associated with Cryptosporidium and Giardia infection is needed to reduce the prevalence of *Cryptosporidium* and *Giardia* infections in < 5 years in Malawi. Furthermore, this knowledge is needed to devise targeted interventions and, thereby, mitigate the adverse consequences of childhood diarrhoea. However, relatively little is known about the epidemiology of these parasites in African countries [6]. In addition, most studies on diarrhoeal diseases in Malawi have focused on national data, while we focused on two health centres in the same area, namely Blantyre, in order to also investigate the heterogeneity within a small area.

The present study aimed to determine the prevalence of *Giardia* and/or *Cryptosporidium* infections and their association with demographic data and anthropometric measurements among < 5 years with diarrhoea attending two primary health facilities in Blantyre (Malawi).

## Methods

### Study area

The research was performed in Blantyre, the second largest city of Malawi, located in the southern part of the country (18_470 N and 98_590 E). The study was implemented in two urban primary health care centres: Ndirande and Limbe. These health centres are located in the densely populated areas of the city. The Limbe health centre has a catchment population of 105,347, while the Ndirande catchment area has 145,187 people who access outpatient health services.

### Study design

This study was a primary health facility-based, cross-sectional study conducted at Ndirande and Limbe health centres run by the Malawian Ministry of Health. The study was performed between February and July 2019, covering a part of the rainy season in which often more diarrhoeal cases are being reported. Approval for the study was obtained from the Research Ethics Committee of the (formerly) College of Medicine (currently Kamuzu University of Health Sciences) (protocol no. P.11/18/2531).

### Study population and data collection

Children < 5 years attending one of the two participating health centres with diarrhoea were recruited for the study. Diarrhoe was defined as more than 3 loose stools in the previous 24 h after a prior interval of 7 diarrhoea-free days [16]. Written informed consent was obtained from the parents or legal guardians on behalf of the child prior to enrolment after explanation of the study objectives either in Chichewa (local language) or in English. A short case record form (CRF) to collect anonymised demographic and basic clinical information about the child was recorded after consent (see below). A formal sample size was not calculated as this was a descriptive study.

### Stool sample collection and diagnostic testing

Stool sample collection was done either directly from diapers, or parents/guardians of recruited children were given labelled stool containers to collect one stool sample on site, after proper instruction. During waiting, children and accompanying adults were provided with drinks and some food and were afterwards transported back to their homes. Only children who were able to produce a stool sample in reasonable time were included in the study.

Collected samples were checked for stool quantity and correct labelling. Fresh samples were analysed immediately on the spot with a RDT for the presence of *Giardia* and/or *Cryptosporidium* infection(s). The RDT used was the GIARDIA/CRYPTOSPORIDIUM QUIK-CHEK (Tech-Lab, USA), which was found to have a suitable diagnostic performance in a previous evaluation [13]. The RDT was conducted at room temperature according to the manufacturer’s instructions. The result of RDT testing was recorded on the CRF.

After testing, all children were referred to an on-site clinician for clinical examination and treatment. The result of diagnostic testing was also communicated to the attending physician. Children who tested positive for *Giardia* were offered treatment with metronidazole. Children who were found to be infected with *Cryptosporidium* were offered supportive treatment with ORS in combination with zinc tablets following Malawi National guidelines.

### Demographic anthropometric data

Basic demographic and anthropometric information of the children with diarrhoea was recorded on the CRF at enrolment. This information included the attending health centre (Limbe/Ndirande), gender (male/female), age (in months) and temperature (in degrees Celsius). To compute stunting and wasting, height (in centimetres) and weight (in kilograms) were obtained from the CRF, which were estimated according to the WHO guidelines [17]. Stunting (no stunting/moderate stunting/severe stunting/outlier) and wasting (no wasting/moderate wasting/severe wasting/outlier) were computed using the World Health Organization (WHO) Anthro Software [18]. Moderate stunting and wasting were defined as having height for age z scores (HAZ) or weight for age z scores (WAZ) <-2 standard deviations below the mean on the WHO Child Growth Standards, respectively [19]. HAZ<-3 and WAZ<-3 were considered severe stunting and severe wasting, respectively. HAZ <-6 or > + 6 and WAZ <-6 or > + 5 were considered outliers and excluded from further analysis [19].

### Statistical analysis

Data from the CRFs were entered and stored in Microsoft Excel spreadsheets and transferred to Statistical Package for Social Sciences (SPSS, IBM Statistics version 28) for processing and analysis. *P* values < 0.05 were considered significant for all statistical analyses performed.

Descriptive statistics of the basic demographic variables were executed to feature the study population and to assess the prevalence of the *Cryptosporidium* and *Giardia* infections. Categorical variables were summarized using frequencies and percentages. For continuous variables, the mean and range were calculated. Furthermore, differences in the basic demographic variables between children negative for *Cryptosporidium* and *Giardia*, children with only *Cryptosporidium*, children with only *Giardia* and children with a coinfection (*Cryptosporidium* and *Giardia*) were assessed using the chi-squared test or Fisher’s-Freeman-Halton exact test for categorical variables and ANOVA for continuous variables. If the assumption of normality was not met for a continuous variable, this requirement was fulfilled by means of transformation of the continuous variable; otherwise, the Kruskal‒Wallis H test was used. When a significant difference between the groups was found for a variable, a Bonferroni correction was applied to counteract the problem of Type I Error that occurs when multiple comparisons are made.

To examine the association between the basic demographic variables and *Cryptosporidium* and *Giardia* infection, simple logistic regressions were conducted. *Cryptosporidium* and *Giardia* were separately analysed, with infection as the dependent variable and the potential associated factors as independent variables. The data from the children with diarrhoea, but without the respective parasitic infection(s) was used for comparison. Participants for whom all basic demographic and anthropometric information was known were included in the analyses. The assumptions for logistic regression were tested before running the analyses. These include independence of errors, linearity in the logit for continuous variables, no multicollinearity, and no strongly influential outliers [20]. The association between the variables and the presence of a *Cryptosporidium* or *Giardia* infection were expressed as univariable odds ratios and their respective confidence intervals.

## Results

### **Prevalence of*Cryptosporidium*and*Giardia*infections**

In total, 972 children < 5 years with diarrhoea were enrolled in this study: 661 cases at Ndirande Health Centre and 311 at Limbe Health Centre (see Table 1). The results of checking the stools of these study cases with RDT for the presence of *Cryptosporidium* or *Giardia* are presented in Table 1. *Cryptosporidium* was detected in 88 stool samples, either as single infection (*n* = 72) or as mixed infection with *Giardia* (*n* = 16), resulting in an overall prevalence of 9.1%. A positive test result for *Giardia* infection was found in 184 participants, either as single infection (*n* = 168) or as mixed infection with *Cryptosporidium* (*n* = 16), resulting in an 18.9% prevalence. Of the 16 participants with a coinfection, one child (6.3%) visited the Limbe health centre, whereas 15 (93.8%) visited the Ndirande health centre.

**Table 1:** Prevalence of *Cryptosporidium* and *Giardia* infections in the Limbe and Ndirande health centres

Variables	Total *n* = 972	Limbe *n* = 311	Ndirande *n* = 661
Only *Cryptosporidium* (*n*, %)			
- No - Yes	900 (92.6) 72 (7.4)	293 (94.2) 18 (5.8)	607 (91.8) 54 (8.2)
Only *Giardia* (*n*, %)			
- No - Yes	804 (82.6) 168 (17.3)	272 (87.5) 39 (12.5)	532 (80.5) 129 (19.5)
Coinfection (*n*, %)			
- No - Yes	956 (98.4) 16 (1.6)	310 (99.7) 1 (0.3)	646 (97.7) 15 (2.3)

### Basic demographic and anthropometric characteristics of the participants

The basic demographic and anthropometric characteristics of the participants are presented in Table 2, distinguishing between children with diarrhoea but who were not infected with *Cryptosporidium* or *Giardia* parasites, children with diarrhoea and only a *Cryptosporidium* infection, diarrhoea with a single infection with *Giardia* and children with diarrhoea and a coinfection (both parasites detected). The outliers of the variables stunting (8/972 (0.8%)) and wasting (4/972 (0.4%)) [19] were excluded from further analysis.

The proportions attending the Limbe and Ndirande health centres were found to be significantly different among the groups (X^2^ (3, *N* = 972) = 16.117; *p* = 0.001). Post hoc comparisons were conducted using a Bonferroni adjustment and revealed a significant difference (*p* = 0.016) in the proportions attending the two health centres between the children with diarrhoea but no *Cryptosporidium* and/or *Giardia* infection (Limbe 35.3%; Ndirande 64.7%) and the children infected with only a *Giardia* infection (Limbe: 23.2%; Ndirande: 76.8%).

The Kruskal‒Wallis H test revealed a significant difference (*H* (3, *n* = 972) = 110.230; *p* < 0.001) in the distribution of age between the groups, and the distribution of age differed significantly among all comparisons, except between negative and only a *Cryptosporidium* infection (*p* = 1.000) and between only a *Giardia* infection and coinfection (*p* = 1.000).

With respect to gender, temperature, stunting and wasting, no significant differences were found between the groups.

**Table 2:** Comparison between the basic demographic and anthropometric characteristics of the study participants who were divided into 4 different groups depending on the outcome of RDT testing for the presence of *Cryptosporidium* and *Giardia* parasites. A significant difference in distribution was only found for the variables: attending health centre and age in months

Variables	Negative for Cryptosporidium and Giardia *n* = 716	Only infection with Cryptosporidium *n* = 72	Only infection with Giardia *n* = 168	Coinfection *n* = 16	*p* value ^a, b^
Attending health centre (*n*, %)					**0.001**
- Limbe - Ndirande	253 (35.3) 463 (64.7)	18 (25.0) 54 (75.0)	39 (23.2) 129 (76.8)	1 (6.3) 15 (93.8)	
Gender (*n*, %)					0.957
- Female - Male	355 (49.6) 361 (50.4)	35 (48.6) 37 (51.4)	85 (50.6) 83 (49.4)	7 (43.8) 9 (56.3)	
Temperature in degrees Celsius (mean, SD)	36.5 (0.8)	36.6 (1.)	36.6 (0.8)	36.2 (0.7)	0.322
Age in months (median, IQR)	14.0 (15.0)	13.0 (12.8)	26.0 (18.5)	24.0 (10.5)	**< 0.001**
Stunting (*n*, %)					0.619
- No stunting - Moderate stunting - Severe stunting	524 (73.7) 120 (16.9) 67 (9.4)	48 (69.6) 15 (21.7) 6 (8.7)	121 (72.0) 36 (21.4) 11 (6.5)	11 (68.8) 4 (25.0) 1 (6.3)	
Wasting (*n*, %)					0.591
- No wasting - Moderate wasting - Severe wasting	645 (90.5) 59 (8.3) 9 (1.3)	64 (90.1) 7 (9.9)	151 (89.9) 14 (8.3) 3 (1.8)	14 (87.5) 1 (6.3) 1 (6.3)	

a) Either Chi-square or Fisher’s freeman exact test for categorical variables; ANOVA or Kruskal‒Wallis H test for continuous variables

b) Bold if significant (*p* value ≤ 0.05).

### **Univariable analyses for factors associated with*Cryptosporidium*and*Giardia*infection**

In Table 3, the results of the univariable analyses examining the association between the basic demographic variables and infection for *Cryptosporidium* and *Giardia* (univariable odds ratio (OR) and respective 95% confidence interval (CI)) are presented. When correcting for outliers for wasting or stunting, 962 out of the 972 participants were included in the analysis. Regarding the potential associated factors, age was not included in the analyses due to violation of the non-multicollinearity assumption (using the matrix of Pearson’s Bivariate Correlation). The variable body temperature was excluded from the analysis due to a high proportion (30.2%) of missing values.

The final potential associated factors taken into the univariable analyses were attending health centre, gender, stunting and wasting. For both infections, the attending health centre was significantly associated with infection in the univariable analysis. Children who attended the Ndirande health centre had 1.8 times higher odds of testing positive for *Cryptosporidium* by RDT than those who attended the Limbe health centre (OR = 1.839, 95% CI: 1.073; 3.152, *p* = 0.027). For *Giardia*, the odds of testing positive were 1.9 times higher in children attending the Ndirande health centre (OR = 1.892, 95% CI: 1.293; 2.767, *p* = 0.001). Neither gender, stunting nor wasting were significantly associated with *Cryptosporidium* or *Giardia* infection in the univariable analyses. Therefore, no multivariable analyses were conducted.

**Table 3:** Results of the univariable analyses of demographic and anthropometric factors of the study participants associated with *Cryptosporidium* and *Giardia* infection

	Cryptosporidium^a^		Giardia^b^	
**Variables**	**Univariable OR (95% CI)** ^**c**^	***p value***	**Univariable OR (95% CI)**	***p*** ** value** ^**d**^
Health centre				
- Limbe - Ndirande	*Reference* 1.839 (1.073–3.152)	**0.027**	*Reference* 1.892 (1.293–2.767)	**0.001**
Gender				
- Female - Male	*Reference* 1.008 (0.645–1.573)	0.973	*Reference* 0.980 (0.710–1.351)	0.900
Stunting				
- No stunting - Moderate stunting - Severe stunting	*Reference* 1.338 (0.775–2.310) 0.980 (0.432–2.220)	0.296 0.961	*Reference* 1.291 (0.865–1.928) 0.711 (0.375–1.347)	0.211 0.296
Wasting				
- No wasting - Moderate wasting - Severe wasting	*Reference* 1.161 (0.539–2.501) 0.950 (0.121–7.456)	0.703 0.961	*Reference* 0.986 (0.549–1.772) 2.136 (0.636–7.180)	0.962 0.220

a. Control group consisting of children with diarrhoea but without *Cryptosporidium* coded as 0, *Cryptosporidium* as 1.

b. Control group consisting of children with diarrhoea but without *Giardia* infection coded as 0, and those without *Giardia* infection coded as 1.

c. OR: Odds ratio, CI: Confidence interval.

d. Bold if significant (*p* value ≤ 0.05).

## Discussion

Infectious diarrhoea is a major cause of mortality in children < 5 years in African populations [1, 2]. The enteric protozoan parasites *Cryptosporidium* and *Giardia* are important causes of diarrhoeal disease worldwide, especially in children < 5 years [5, 6]. Therefore, adequate knowledge about their epidemiology is of importance. The present study aimed to determine the prevalence of *Cryptosporidium* and *Giardia* infection and their association with demographic or anthropometric measurements in Blantyre, Malawi. The *Cryptosporidium* spp. prevalence in children under five with diarrhoea in the present study was found to be 9.1%. This is higher than the prevalence reported in another study from the same region, where the prevalence in paediatric children under five was found to be 5.9% [21]. However, a more recent case‒control study conducted in 2019 in children under five in Blantyre hospitalized with diarrhoea reported a prevalence of 27.8% [22]. Regarding *Giardia*, the prevalence in the present study was 18.9%. This is more than two times higher than a similar study conducted in Blantyre, Malawi, where the prevalence in stool samples of children under five with diarrhoea was found to be 7.3% [22]. A possible explanation for these differences in prevalence might be due to the variations in methods used for detection, microscopy (5.9%) [21] vs. PCR (27.8%) [22] vs. RDT (9.1%; this study) as well as the different time periods in which samples were collected (rainy season) and the fact that the present study population comprised out patients only and not hospitalised cases.

It is of interest to note that although both *Cryptosporidium* and *Giardia* have the same mode of transmission and share habitats, they have equal opportunity to infect children. Nevertheless, the number of coinfections was low. Only 16 (1.6%) children of the total study population had a *Cryptosporidium* and *Giardia* coinfection. Among the children positive for *Cryptosporidium*, 18.2% (16/88) were coinfected with *Giardia*. This is in line with several other studies that reported low to no coinfections of these parasites [23–25]. In contrast, other studies have reported a much higher prevalence of coinfections, ranging from 8.7% to almost 30% [26, 27]. A reason for this discrepancy is likely location dependent.

With respect to the association between parasite infection and demographics, it was found that attending health centres were significantly associated with both *Cryptosporidium* and *Giardia* infection. Children who attended the Ndirande health centre had a higher odds of testing positive by RDT for both infections. This is substantiated by the prevalence of *Cryptosporidium* and *Giardia*, which were both found to be higher in children attending the Ndirande health centre in comparison to those attending the health centre in Limbe. An explanation for this positive association might be that Ndirande is more densely populated and houses are even more closely clustered together.

Regarding the age of the participants, children with only a *Giardia* infection had a median age of 26 months and were found to be significantly older than the children with a *Cryptosporidium* single infection, who had a median age of 13 months. The latter is supported by the finding of the GEMS study, which reported that Cryptosporidium is the second most important cause of diarrhoea in children under two years of age. In the GEMS study, however, no association between *Giardia* infection and age was found [16]. Nonetheless, both ages are vulnerable to diarrhoeic diseases because they may lack knowledge about basic hygiene rules or fail to adhere to them, but this does not explain the observed age difference and further behavioural research might be needed to underpin the underlying cause for this.

No significant association between stunting and infection or between wasting and infection was found for either *Cryptosporidium* or *Giardia*. These findings contradict with other studies, which found a negative association between *Cryptosporidium* and *Giardia* infection and length and weight [28–30]. Enteric protozoan parasites are in general associated with lower growth in young children, regardless of diarrheal symptoms, with *Cryptosporidium* and *Giardia* being associated with growth shortfalls among asymptomatic children [31]. Furthermore, *Cryptosporidium* infection, amongst other enteric pathogens, was associated with growth faltering in children with diarrhoea [32]. Regarding gender, no significant association with *Cryptosporidium* or *Giardia* infection was found, which is supported by previous studies conducted in different countries [33–35].

In 26.3% (256/972) of the diarrhoea cases, a protozoan parasitic infection was the likely cause of disease. For those cases with *Giardia* infection, treatment with metronidazole is indicated, and in general for diarrhoeal cases (including for those with *Cryptosporidium* or *Giardia* infection), supportive treatment with ORS and zinc is indicated. There might have been an underestimation of the actual number of cases infected with *Giardia* and/or *Cryptosporidium* because of the inherent limitation in sensitivity of the used RDT. This leaves a substantial population undiagnosed, and the aetiology of diarrhoea in these children remains unknown. Infections with rota-, adenovirus, and norovirus are other main causes of infant diarrhoea in Blantyre [22, 36, 37]. Despite the introduction of a vaccine against rotavirus and sustained high coverage of this vaccine in Malawi, the burden of rotavirus disease remains high and significantly contributes to the number of cases [38].

Our study was limited by the fact that we have used an RDT to detect the *Cryptosporidium* and/or *Giardia* in the stool samples of the children with diarrhoea. Molecular methods, such as PCR, are considered to be more sensitive to detect these infections [13], and we might thus have missed some cases. However, the settings we worked in did not allow implementation of PCR due to infrastructural constraints. Moreover, we needed to have the result of testing available as soon as possible as this was an outpatient study and cases were not hospitalised. In such a setting the use of RDT is warranted and the test used in the current study had to our opinion a good diagnostic performance [13].

The study has explored the presence of *Cryptosporidium* and/or *Giardia* only. Other enteric pathogens have not been taken into account and these may significantly contribute to diarrhoea and its effect on child health [31, 32]. This is confirmed by the fact that 72.0% of the children with diarrhoea were not found to be positive for either *Cryptosporidium* or *Giardia* or both. Future studies are required on the role of other entero-pathogens in the ethiology of diarrhoea in our setting.

## Conclusion

*Cryptosporidium* and *Giardia* infections are highly prevalent in < 5 years with diarrhoea attending primary health centres in Blantyre, Malawi. Children with only a *Giardia* infection were found to be significantly older than the children with a *Cryptosporidium* single infection. In our study, parasite infection seemed not to be associated with gender, stunting and wasting. The role of other entero-pathogens should be taken into account in future studies too.

## Data Availability

The data used to support the findings of this study are available from the corresponding author upon request.

## Abbreviations

CRF: Case record form
HAZ: Height for age z scores
ORS: Oral rehydration solution
RDT: Rapid diagnostic test
SPSS: Statistical Package for Social Sciences
WAZ: Weight for age z scores
WHO: World Health Organization
